# Successful Management of Multiple Giant Anterior Sacral Meningoceles Through an Anterior Approach in a Patient With Marfan Syndrome: A Case Report and Literature Review

**DOI:** 10.7759/cureus.52724

**Published:** 2024-01-22

**Authors:** Anis Choucha, Sacha Tomczak, Nicolo Norri, Jean Hardwigsen, Henry Dufour

**Affiliations:** 1 Neurosurgery, Assistance Publique-Hopitaux de Marseille (AP-HM), Marseille, FRA; 2 Laboratory of Biomechanics and Application, Gustave Eiffel University, Aix Marseille University, Marseille, FRA; 3 Plastic and Reconstructive Surgery, Assistance Publique-Hopitaux de Marseille (AP-HM), Marseille, FRA; 4 Neurosurgery, University of Ferrara - Sant'Anna Hospital, Ferrara, ITA; 5 General Surgery, Assistance Publique-Hopitaux de Marseille (AP-HM), Marseille, FRA

**Keywords:** meningoceles, csf leak, omental flap, marfan, anterior sacral meningoceles, case report

## Abstract

Meningoceles refer to the protrusion of meninges filled with cerebrospinal fluid (CSF) through a bone defect. There is scarce literature on the management of multiple giant anterior sacral meningoceles (ASMs). We report the case of a patient with Marfan syndrome presenting with gait disturbances and dizziness triggered by posture changes due to multiple giant ASMs. The patient was managed through an anterior approach involving a multidisciplinary team of surgeons. Care was taken to limit the persistence of CSF leak using an omental pedicled flap. This technique has only been mentioned twice in the literature for such cases. A literature review was conducted focusing on the evolution course and surgical strategy of meningoceles.

## Introduction

Meningoceles are cerebrospinal fluid (CSF)-filled protrusions of meninges through a bone defect [1]. Anterior sacral meningoceles (ASMs) are mainly associated with underlying connective tissue disorders such as Marfan syndrome [2,3]. ASM usually manifests as constipation, urinary disturbances, irregular menstruation, headaches, and lower limb weakness or pain. Giant symptomatic ASM requires a multidisciplinary team of surgeons for management. Postoperative CSF leak is a prime concern and must be carefully addressed in surgical planning. Here, we report the successful management of a patient with Marfan syndrome presenting with multiple giant ASMs through an anterior approach with the use of an omental flap to prevent postoperative CSF leak.

## Case presentation

History and examination

A 60-year-old female patient with Marfan syndrome presented to our facility with gait disturbances, dizziness, and instability triggered by posture changes. A few months earlier, after a boat trip where the patient sat at the bow of the boat with ascending and descending motion, she reported severe bilateral sciatica which spontaneously resolved. She was a known case of ASM, incidentally diagnosed 20 years earlier and followed up regularly.

On clinical examination, the patient had a poorly defined bilateral lower limb weakness with no pyramidal sign, absent Achilles reflex, and hyperactive knee jerks. The Lasègue sign was positive 45° bilaterally. She reported urinary and bowel dysfunction with morning dysuria followed by urinary incontinence during the day and constipation. She suffered from episodic headaches. A firm palpable cough-impulse mass was noted on the abdominal examination.

Twenty years ago, an MRI had revealed four anterior sacral meningoceles protruding through the right and left S1 and S2 foramen. As these were considered incidental findings, a wait-and-see policy was adopted, and the patient was followed up regularly. Her mother, also suffering from Marfan syndrome, was operated on for a giant ASM 20 years earlier.

**Figure 1:**
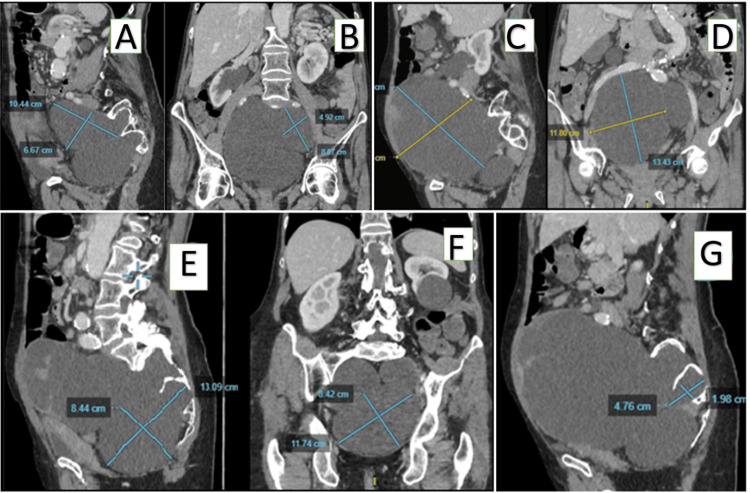
Preoperative abdominal CT scan. Sagittal (A) and coronal (B) imaging of the left S1 meningocele (11 x 5 x 5 cm), sagittal (C) and coronal (D) imaging of the right S1 meningocele (12 x 15 x 16 cm), sagittal (E) and coronal (F) imaging of the left S2 (10 x 11 x 8 cm), and sagittal imaging of the right S2 meningocele (G) (3.7 x 3.5 x 3.2 cm). Meningoceles described are outlined with white arrows.

A new MRI was not feasible as the patient had a pacemaker due to cardiac complications of her condition. Thus, a pelvic CT and a pelvic radiculography were performed. It showed that the four meningoceles had grown significantly (Figure 1). They now caused severe bladder compression and shifted the right ureter and the rectum forward (Figure 1).

Operation

**Figure 2:**
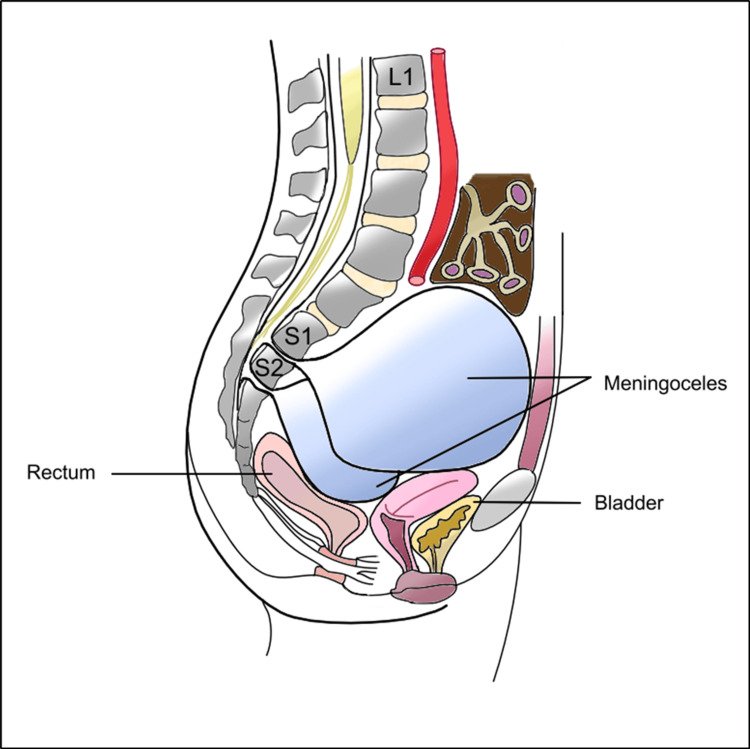
Illustration of the meningoceles with relevant surrounding anatomical structures.

**Figure 3:**
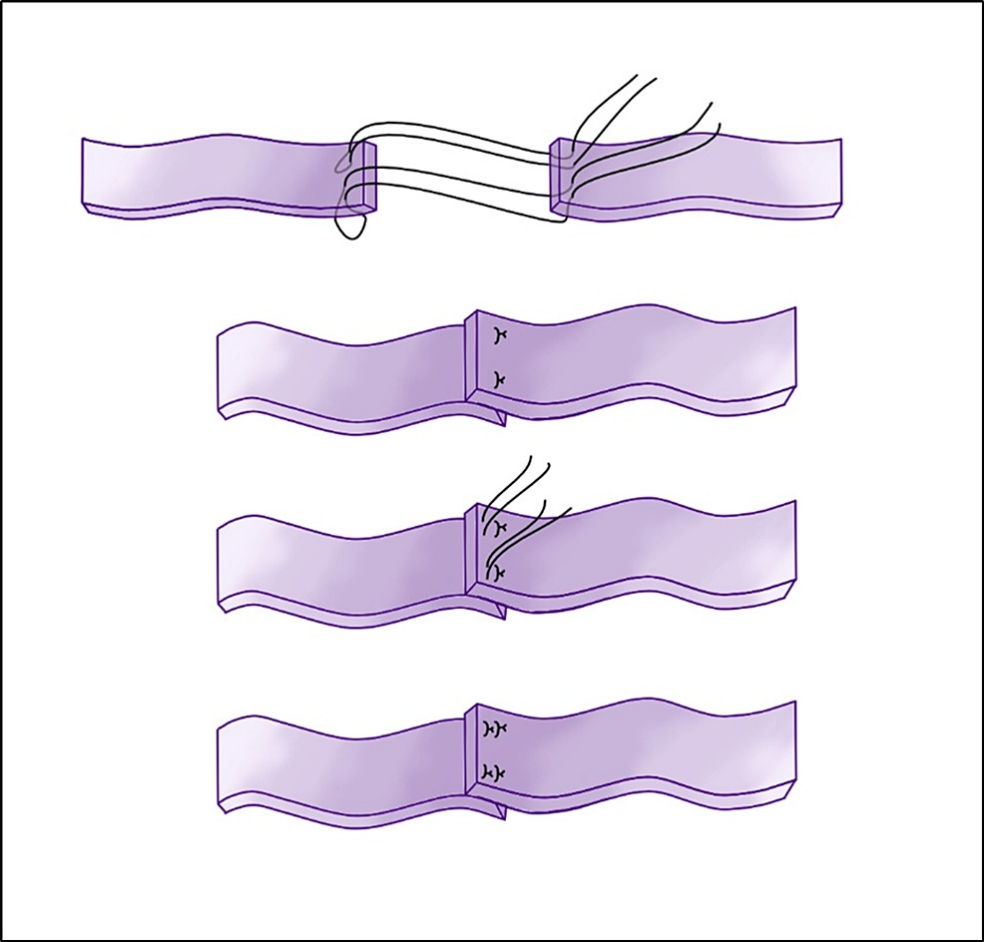
Illustration of the closure of the remaining dura mater in a so-called “paletot trend.”

With the assistance of a colorectal surgeon, an anterior transperitoneal approach was used (Figure 2). The patient was positioned supine and a median vertical incision above the pubis was made. A standard laparotomy was performed, giving access to the peritoneal cavity. The incision was deepened, exposing the biggest meningocele which rose from the pelvis, shifting the bowels upward. Adhesiolysis and blunt dissection provided direct access to the anterior walls of the meningoceles. Incision of the dural wall revealed xantochromic fluid and hematoma. This turned out to be an intracystic hemorrhage creating a false wall on this cyst. Evacuation of this hematoma unveiled an underlying wall that was incised. Drainage of the meningocele associated with careful and precautious dissection of the nearby pelvic organs allowed reaching a widely dilated right S1 foramen. The wall was addressed to the pathology department and the remaining dura mater was sutured in a paletot trend (Figure 3). Emptying the biggest meningocele led to the collapse of the remaining three others as they communicated through the meninges. To spare important vessels such as the hypogastric veins or sacral plexus, we adopted a function-sparing strategy. Hence, no further surgery was performed on the right S2 meningocele and left S1 meningocele, which collapsed. On the other hand, the left S2 meningocele was still prominent and free from essential vessel adherence; hence, it was removed and stitched in the same trend as the right S1. Finally, a pedicled omental flap was separated from the stomach and tacked adjacent to the dural defect in the anterior sacrum, filling the ASM sac to prevent further leakage or re-expansion.

Postoperative course

**Figure 4:**
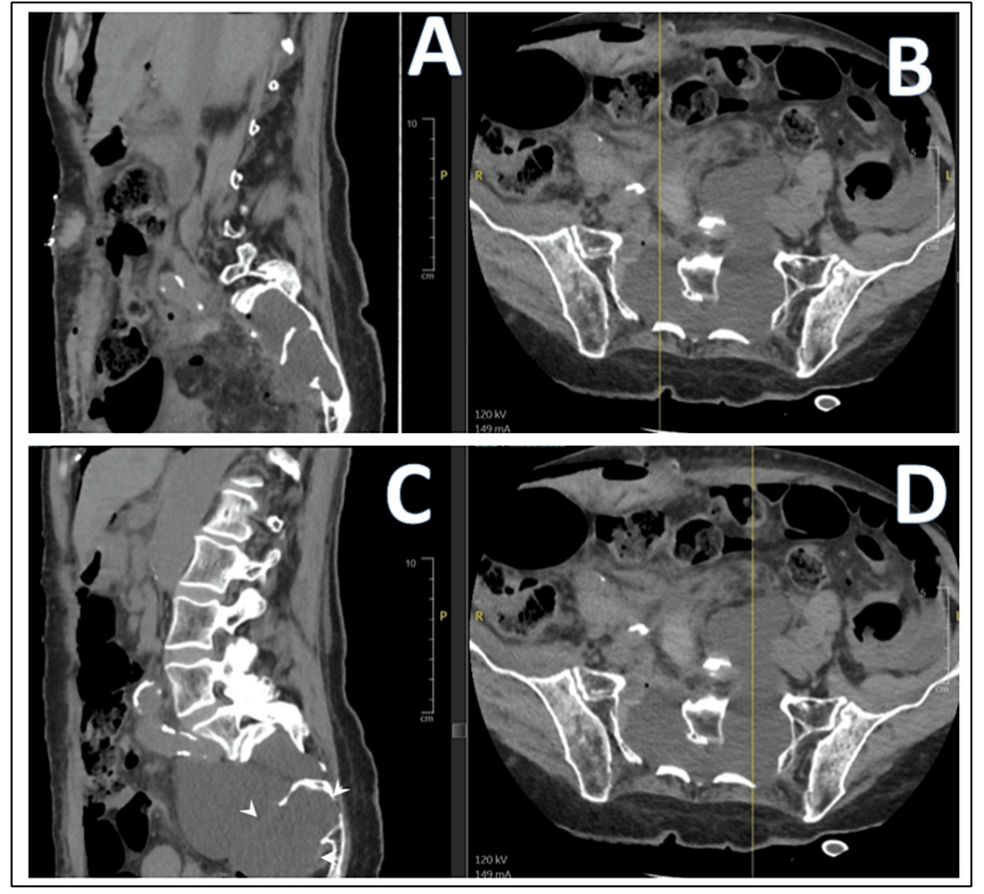
Postoperative abdominal CT. Sagittal (A) and axial (B) images focusing on the right upper sacral meningocele, and sagittal (C) and axial (D) images focusing on the left lower meningocele.

Afterward, the patient was admitted to the intensive care unit. She was maintained in a Trendelenburg position for four days. A nasogastric tube was inserted, and the bladder catheter was kept postoperatively. The first two days were uneventful, allowing a transfer to the neurosurgical department. The Trendelenburg position was maintained postoperatively until day four. That same day, the nasogastric tube and the bladder catheter were withdrawn. The control CT showed a successful resection of the right S1 meningocele and a significant regression of the left S2 meningocele (Figure 4).

The patient recovered full autonomy, was able to walk, and bowel function was retrieved with laxatives. A post-obstructive diuresis syndrome led to a reinsertion of a bladder catheter. On postoperative day 12, the bladder catheter could be weaned off. The patient complained about orthostatic headaches controlled through paracetamol. She was discharged on the 14th postoperative day. Pathology examination revealed fibrous and inflammatory tissue with pseudo-slits and no epithelium. Orthostatic headaches were absent at the follow-up appointment (day 45 postoperative), and so were the abdominal discomfort and constipation. The patient was free from gait disorder. As her post-void urinal residue was 200 cc, she was taught to use self-catheterization twice daily. At the one-year follow-up, the patient remained neurologically intact.

## Discussion

Definition, prevalence, and pathophysiology

Meningoceles are congenital or acquired CSF-filled protrusions of meninges (both dura mater and arachnoid) through a bone defect or the enlargement of a spinal foramen [1]. They differ from myelomeningoceles, which also contain neurological tissue, and pseudo-meningoceles which consist of CSF collections without surrounding meninges (mostly post-traumatic or iatrogenic to surgery) [1]. Although ASM following minor trauma has been described, it is mainly associated with underlying connective tissue disorders such as neurofibromatosis type 1, Ehlers-Danlos syndrome, Currarino syndrome, and, in our case, Marfan syndrome [2,3].

In sporadic cases, ASM is most likely due to a congenital neural tube abnormality arising during embryonic development (neurulation stage), inducing a defect in the anterior sacral wall. In Marfan syndrome, the development of ASM is caused by regular CSF pulsation on predisposed fragile dural and bone tissue, resulting in progressive growth and erosion of the sacral wall [4,5]. CSF pressure is most significant in the caudal regions of the spinal canal because of orthostatism, which explains why the sacral region is most frequently impacted. Indeed, with a prevalence of 1/10,000, ASM accounts for 20% of cases [6]. Bryant first described ASM in 1837 [7].

Clinical and radiological features

In young patients, because of their small size, ASMs are usually asymptomatic. During the evolution course, ASM growth can exert pressure on surrounding organs, causing compression of the urinary tract, rectum, uterus, or sacral nerve roots, which manifest as lower urinary tract symptoms, chronic constipation, irregular menstruation, and pain or weakness in lower limbs [8-10].

Furthermore, intermittent changes in intracranial pressure can lead to orthostatic headaches or headaches related to Valsalva maneuvers, as reported in 10-15% of cases [11]. Baldia et al. reported the case of a teenager who could not withstand the prone position due to subsequent headaches [12].

Diagnosis is made using MRI, which allows differentiating ASM from myelomeningocele and pseudo-meningoceles. Moreover, it provides accurate information about the size, shape, anatomical relationships with surrounding organs, and intrinsic characteristics of cystic masses. In this case, an MRI was performed at the time of diagnosis but was not feasible currently. Hence, we performed a contrast-enhanced abdominal CT associated with lumbosacral radiculography. Other tests, such as urinary pressure flow study, may help assess preoperative symptoms.

Natural history and surgical management

Asymptomatic ASM can be managed conservatively. Nevertheless, no spontaneous regression of ASM has been reported in the literature. Furthermore, life-threatening complications, such as fistula [13] and meningitis [14], have been described. Thus, patients must be informed of the potential evolution course and the prospect of surgery later on. A follow-up with iterative clinical evaluation and imaging is indicated in such cases. If the lesion turns symptomatic, or to prevent complications in case of a growing lesion, a surgical procedure is indicated. Decision-making requires a multidisciplinary team with a neurosurgeon, colorectal surgeon, urinary surgeon, or general surgeon. Risks and benefits should be balanced carefully as the surgical procedure carries risks, for instance, persisting CSF leakage, abdominal organ lesion, infection, sacral plexus injury, and recurrence.

The goals of the surgery are to isolate the meningocele from the sacral subarachnoid space circulation, relieve pelvic organ pressure by removing the tumor, and, in some cases, untether the spinal cord or treat other associated disorders.

Several surgical approaches have been described in the literature, including anterior transabdominal and posterior sagittal approaches [5,8-10]. Although transrectal and transvaginal aspirations have been used in the past, these options have proven detrimental due to risks of infection and recurrence [15].

The posterior sagittal approach uses a midline incision that runs from the sacrum to the coccyx. Through a laminectomy, the meningocele is reached. Then, the strategy consists of draining the meningocele and obliterating the communicating holes from the subarachnoid spinal space. The cyst then resolves over time. Moreover, in other cases, this type of approach allows the treatment of associated conditions when present, such as untethering the spinal cord, obliterating an anorectal fistula, or removing the dermoid cyst.

Because of these advantages, the surgical team carefully contemplated a sagittal posterior approach. However, because of the size of the ASM and the need to relieve pressure from the meningoceles in the abdominal cavity, an anterior approach was preferred. In our case, meningoceles were not associated with other abnormalities. Moreover, this surgical strategy allows removing the cyst, relieving pelvic compression instantly. Hence, we concluded that the benefits of an anterior approach outweighed the risks and the patient agreed with this strategy. With the help of a colorectal surgeon, we accessed the lesion with broad exposure and safely dissected neurovascular structures.

Limiting the risks of CSF leaks is a prime concern during surgical planning. In our case, we used a paletot trend to close the dural stalk rather than a watertight running suture, which we believe lowered the risks of CSF leakage [16]. On top of that, we added an omental flap covering the suture. The omental flap has various applications in reconstructive surgery. Its molecular properties promote healing at recipient sites [17]. In the last decade, omental flap has found its way into the management of complex cases of CSF disorders. Omental flaps have been described in the management of chronic CSF leaks following a transsphenoidal approach or to cure complex spinal wounds [17,18]. Furthermore, Levander et al. published the case of a patient with severe chronic communicating hydrocephalus with several successive shunt malfunctions who was finally successfully treated with a lumbar-omental shunt [19]. To our knowledge, this case is the third in the literature reporting the successful use of an omental flap in managing anterior sacral meningocele, after Bergeron et al. and Paisan et al [10-20].

Postoperatively, the patient was kept in a Trendelenburg position. It was maintained until the fourth postoperative day. This adjuvant therapy was used to lower the risks of CSF leaks [12]. This is based on the findings of an experimental dural healing study, which found that the primary fibroblastic bridging of the durotomy starts by day six and is completed by day 10. The lumbar subarachnoid CSF pressure is higher in the sitting and standing positions and the lowest in the supine position with foot end elevation. While there are very few studies on the effect of maintaining Trendelenburg position after surgery involving the opening of the spinal dura, its use has been widely reported to prevent risks of CSF leak.

Other preoperative measures have been mentioned in the literature, which include the use of preoperative colic preparation or the use of an external lumbar drain. We chose not to use the latter because of the theoretical risk of CSF leak and intracranial hypotension afterward due to the underlying Marfan syndrome in our case.

We believe this case is interesting for many reasons. First, giant ASM remains a rare entity in the literature requiring individualized treatment plans. Furthermore, this case has some singularities: both the patient and her mother were operated on for ASM, and the patient presented a hematoma inside the wall of the meningocele, which could be linked to the episode of bilateral sciatica that followed her boat trip with ascending and descending motion a few months before. Only one case of such a hematoma has been reported in the literature [8]. Further, most ASMs are managed via a posterior approach which contrasts with our patient’s management. Lastly, we report the successful use of an omental flap. Only two cases in the literature have reported using an omental flap in this context. We believe that the addition of the omentum covering the suture helped watertight the closure.

## Conclusions

ASM is a rare entity that may require surgery. The procedure involves a multidisciplinary team and a tailored surgical strategy. Although a posterior approach is more commonly used, an anterior approach must be considered for giant ASMs exerting pressure on surrounding structures. In such cases, preventing CSF leaks is a primary concern. A pedicled omental flap seems to be a valuable tool for achieving a good clinical result.
